# Microbiome of Bovine Milk and Factors Influencing Its Composition

**DOI:** 10.3390/ani16070996

**Published:** 2026-03-24

**Authors:** Łukasz Szala, Justyna Staninska-Pięta, Agnieszka Piotrowska-Cyplik

**Affiliations:** Department of Food Technology of Plant Origin, Faculty of Food Science and Nutrition, Poznan University of Life Sciences, Wojska Polskiego 31, 60-624 Poznan, Poland; justyna.staninska@up.poznan.pl (J.S.-P.); agnieszka.piotrowska-cyplik@up.poznan.pl (A.P.-C.)

**Keywords:** Bovine milk microbiome, bovine mammary microbiome, molecular biology, metagenomics, raw milk safety, 16S rRNA sequencing, diary processing, lactation stage, review

## Abstract

The bovine milk microbiome is a complex and dynamic microbial ecosystem, comprising both commensal and pathogenic bacteria. Its composition is shaped by endogenous factors, including udder physiology, lactation stage, and health status, particularly mastitis, as well as by exogenous factors, such as housing conditions, farm infrastructure, milking practices, and post-milking processing. Mastitis not only alters milk quality but also induces persistent dysbiosis that may persist even after clinical recovery, highlighting the need for continuous microbiome monitoring to ensure milk safety. Advances in molecular and metagenomic techniques have enabled the detection of microbial taxa that are difficult to identify using traditional culture-based methods. However, challenges remain due to low microbial biomass, reagent contamination, and the inability to distinguish live from dead bacteria, all of which complicate accurate characterization. Environmental contamination from skin, air, and equipment, along with microbial shifts during transport, storage, pasteurization, and product separation, further modulate microbial communities. While mastitis-related changes in milk microbiota have been extensively studied, the effects of other bovine diseases and systemic health conditions remain largely unexplored, constituting a critical knowledge gap. Understanding the factors that shape milk microbial communities is essential for ensuring dairy product safety, optimizing herd management, and developing microbiome-based innovations in milk production.

## Simple Summary

Most bacteria present in nature cannot be detected using traditional laboratory culture methods, and for many years, milk was believed to be almost sterile. Modern DNA-based techniques have revealed that cow’s milk contains a complex and changing community of microorganisms. However, scientists still do not fully understand which bacteria truly belong to milk and which are contaminants introduced during milking, storage, or laboratory analysis. The aim of this study was to summarize current knowledge about the milk microbiome and to identify the key factors that shape it. The analysis shows that milk microorganisms are influenced by cow health, especially udder inflammation (mastitis), as well as housing conditions, milking equipment, storage, and milk processing steps such as cooling and pasteurization. Because research methods are not yet standardized, comparing results between studies remains difficult. Understanding how and when milk becomes contaminated, and which microorganisms are naturally present, is essential for improving food safety, extending shelf life, supporting animal health, and developing better dairy production practices that benefit farmers, consumers, and public health.

## 1. Introduction

It is estimated that approximately 99% of microorganisms present in the environment cannot be identified using traditional culture-based methods. The development of molecular techniques and DNA sequencing has enabled increasingly profound, rapid, and cost-effective insights into the microbiome of the surrounding world, including milk [1]. The first metagenomic studies on milk microbiota were conducted in 2012, and since then, a growing number of researchers have sought to investigate the milk microbiome. Although these emerging analyses provide ever more information on the diversity of bacteria in milk and the factors that influence them, many aspects remain unknown. Of particular importance was the discovery that the milk cistern environment is not sterile, and that it may be inhabited by certain groups of microorganisms [2].

Despite two decades of research into bacteria inhabiting milk, standardized analytical and sampling methods have not yet been established. This limits the comparability of studies and leads to inconsistent conclusions. The milk microbiota appears to be a dynamic environment, whose diversity depends on geographical location [3], post-milking and technological operations [4,5], rearing systems [6], as well as the milk sampling method [7], among many other known and unknown factors. Due to these factors, defining the “core microbiome” of milk remains a difficult task, although such attempts have been made [8].

This paper aims to present the current state of knowledge on the microbiome of cow’s milk and to discuss the key factors influencing its composition. This analysis will identify the relationships among farming practices, milk processing conditions, and microbiological quality, which are of significant importance for both public health and the efficiency of milk production. Furthermore, understanding the mechanisms shaping the milk microbiome may provide a basis for implementing strategies to improve the safety of dairy products and to support the development of innovative technologies for assessing the milk microbiome.

## 2. Core Microbiome and Analysis Challenges

Milk, due to its high water-activity, proteins, carbohydrates, fats, vitamins, and minerals, constitutes an environment conducive to the growth of microorganisms, both pathogenic and beneficial to human health [9]. It is estimated that 1 mL of milk may contain between 10^3^ and 10^5^ CFU of bacteria, exhibiting very high species diversity [10]. Research on the milk microbiome and the microbiota of dairy products is a rapidly developing field, and the increasing use of modern molecular biology techniques continues to expand our understanding of this ecosystem.

Defining the core milk microbiome remains challenging because determining the appropriate sampling stage is difficult. Studies indicate that microbial composition varies depending on whether samples are collected directly from the udder, the teat canal, bulk tanks after transport, or during storage and delivery to processing facilities [4,11,12].

In previous studies, milk obtained from healthy heifers most frequently contained bacterial genera such as *Faecalibacterium*, *Lachnospiraceae*, *Propionibacterium*, *Aeribacillus* [13], *Pseudomonas*, *Ralstonia* [14], *Psychrobacter*, *Bacillus*, *Enterococcus*, *Lactococcus*, *Halomonas*, *Clostridium*, *Staphylococcus*, *Lysinibacillus*, *Streptococcus*, *Marinilactibacillus*, *Lactobacillus*, *Vagococcus*, *Alkaliphilus*, and *Macrococcus* [15].

Metagenomic analyses of raw milk from healthy cows revealed the presence of bacteria belonging to Firmicutes, Proteobacteria, Bacteroidetes, and Actinobacteria [11,16,17]. However, it should be noted that the milk microbiome of healthy cows is highly variable, which makes it difficult to define its basic commensal core due to the lack of standardized procedures for sample collection, processing, and analysis [18].

The interpretation of the presence of *Pseudomonas*, *Halomonas*, and *Shewanella* in milk microbiome datasets remains controversial, as their detection may arise from the contamination of laboratory reagents, particularly DNA polymerases and extraction kits. Airborne microorganisms within biosafety cabinets or laboratory environments may also represent a non-negligible source of exogenous DNA contamination. Given the inherently low microbial biomass in milk and the resulting low yield of isolated DNA, even minor reagent- or environment-derived contaminants can substantially influence sequencing outcomes and bias microbiome composition analyses [7,19]. Initially, milk accumulating in the udder cistern was considered inherently sterile, a view derived from traditional culture-dependent methods [2]. However, culture-independent molecular analyses have not provided conclusive evidence to refute this assumption. Ref. [7] conducted genetic analyses of milk collected from individual udder quarters and directly from the cistern, observing the highest DNA yield and amplification success in cisternal samples. Nevertheless, bacterial growth could not be achieved from these samples, suggesting that the detected DNA may have originated from bacterial remnants processed by immune cells or from low-level environmental contamination. The study thus highlighted the cistern’s ambiguous role as a potential microbial reservoir and underscored the need for further investigation.

## 3. Host-Related Factors Shaping the Bovine Milk Microbiome

The bovine milk microbiome is a dynamic and complex environment shaped by both host- and non-host-related factors. Host-related determinants include udder physiology and health status, encompassing both physiological processes in healthy animals and pathological conditions that may disrupt microbiome balance. Non-host-related factors include rearing systems, environmental conditions, and technological milk handling and processing.

### 3.1. Stage of Lactation

The stage of lactation is an important endogenous factor influencing milk microbiome composition. Colostrum harbors a diverse and distinct microbiome, including a core set of mammary-associated bacteria, and its composition varies with parity and udder health status, highlighting early lactation as a critical and dynamic period for microbiome establishment [20]. Early lactation is a biologically important period during which the milk microbiome is highly dynamic and may be relevant to both mammary gland health and the calf’s early microbial exposure. Ref. [21] highlighted early lactation (0–15 days postpartum) as critical for microbiota establishment, showing higher abundances of gut-associated genera (*Lactobacillus*, *Bacteroides*) in the early lactation stage. Colostrum is a critical source of beneficial microbes for neonatal calves, contributing significantly to the early establishment of gut microbiota. Recent studies have identified dominant taxa in bovine colostrum, including *Paraclostridium*, *Romboutsia*, and *Staphylococcus*, as well as major phyla such as *Pseudomonadota*, *Bacillota*, and *Bacteroidota*. A substantial proportion of species found in neonatal feces on day one are derived from colostrum, many of which persist in the gut for weeks, underscoring colostrum’s dual role as a nutritional source and a key vector for early microbiota establishment that may impact calf health and development [22]. Previous studies by [20] identified a core microbiome including, among others, *Staphylococcus*, *Prevotella*, *Ruminococcaceae*, and *Bacteroidales*. Colostrum microbiota composition can vary between cows and is influenced by factors such as parity and host genetics, with primiparous cows and certain BoLA genotypes exhibiting higher microbial richness and diversity, while these genotype-associated effects diminish in milk collected later in lactation [20,23]. Moreover, variability across studies, driven by factors such as farm management, breed, environment, and methodological differences, limits the generalizability of core microbiome findings.

Microbial diversity and composition continue to change over the first two months postpartum, with primiparous cows exhibiting more pronounced temporal shifts com-pared to multiparous cows, and gut-associated taxa fluctuating in abundance during this period [23]. The transition from colostrum to milk is associated with significant increases in microbial diversity during the first week of lactation, indicating dynamic shifts in the microbiome during the early postpartum period [23,24]. Ref. [25] identified significant associations between specific bacterial taxa and total protein content in bovine milk. This suggests that microbial community structure may be linked to milk protein dynamics, potentially reflecting interactions between milk composition and mammary metabolism.

Due to limited available data, further research is needed to better understand the milk microbiome. Gaining insight into its temporal evolution throughout lactation and host-associated variability is essential for elucidating its functional role and potential implications for dairy management and neonatal development.

### 3.2. Udder Cistern Microenvironment

Initially, milk accumulating in the udder cistern was considered inherently sterile, a view derived from the use of traditional culture-dependent methods [2]. However, the application of culture-independent genetic analyses has not provided conclusive evidence to contradict this assumption. Refs. [7,26] performed genetic analyses of the milk microbiome collected from individual udder quarters and directly from the milk cistern. Their results indicated that milk obtained from the cistern showed the highest likelihood of successful bacterial DNA amplification and the greatest DNA yield among all tested variants. Nevertheless, these results have not been consistently confirmed in subsequent studies [27]. This inconsistency highlights the need to systematically report this parameter in future research to facilitate methodological standardization and the comparability of results. The authors noted that they were unable to culture bacteria from samples taken directly from the cisterns. Therefore, the origin of the detected genetic material cannot be definitively established. The amplified DNA might have originated from bacterial remnants processed by immune cells present in the milk environment. The study highlighted the ambiguous role of the cistern as a potential microbial reservoir and emphasized the need for further research [7]. These findings underscore the importance of molecular methods in milk microbiome studies, especially for detecting viable but non-culturable (VBNC) microorganisms, and illustrate that genetic analyses do not always reflect the actual microbial composition of the environment under study. It has also been noted that although the teat canal serves as a natural barrier preventing bacterial entry into the udder, certain conditions, such as sphincter relaxation after milking, may weaken this defense, increasing susceptibility to infections by both pathogenic (e.g., mastitis-causing) and commensal bacteria such as lactic acid bacteria [16]. Another limitation in the comprehensive characterization of the milk microbiome is the specificity of the analytical methods employed. In many studies, amplification of selected regions of the 16S rRNA gene, most commonly V1–V4, is performed before DNA sequencing. While this approach is cost-effective, it may overlook low-abundance taxa, thereby limiting the complete depiction of microbial diversity. An alternative strategy is shotgun metagenomics, which enables analysis of the entire genomes of the microorganisms present and provides information on the metabolic functions of individual genes. However, this method is associated with higher costs and greater analytical demands. Therefore, when designing studies on the milk microbiome and interpreting the results, it is essential to consider the limitations and capabilities of the employed methodology.

### 3.3. “Rumen–Mammary” and “Entero-Mammary” Pathways

Although external factors are considered dominant in shaping the milk microbiome, recent studies have also indicated the significance of internal determinants, including the physiology of the mammary gland [28]. The presence of anaerobic bacteria in raw milk supports the influence of endogenous factors. It is hypothesized that such microorganisms may colonize milk via internal systemic pathways known as the “gut–udder” and “rumen–udder” routes.

The entero-mammary pathway is presumed to involve phagocytosis. This hypothesis suggests that intestinal monocytes, dendritic cells, and macrophages may engulf bacteria and transport them through the tight junctions of the intestinal epithelium without disrupting its integrity or destroying the internalized microbes. These immune cells can then migrate via mesenteric lymph nodes or directly through the circulatory–lymphatic system to the mammary gland, where potential colonization may occur. While this mechanism has been described in humans, it remains hypothetical in bovines [29,30]. The detection of taxa such as *Ruminococcus*, *Bifidobacterium*, and members of Peptostreptococcaceae in both feces, blood leukocytes, and milk of the same cows suggests a possible translocation of bacterial components or, less likely, viable cells via an immune-mediated pathway [31].

The rumen–mammary pathway is less understood. The rumen is a microbially rich environment containing protozoa, viruses, fungi, and bacteria. It is estimated that 1 mL of rumen fluid contains approximately 2.7 × 10^9^ CFU of bacteria, while 1 g of solid rumen content contains 5.6 × 10^11^ CFU. In milk samples collected from quarters affected by mastitis, bacterial genera such as *Oscillospira*, *Clostridium*, and *Selenomonas* were detected. However, their genetic material accounted for less than 1% of the total identified sequences. These genera are also found in the rumen, supporting the hypothesis of their possible migration through the rumen–mammary pathway. Although further research is required, the compositional similarity between rumen and milk, as well as the shared presence of certain anaerobes, lends credibility to this concept [25,30].

### 3.4. Mastitis and Milk Microbiome Dysbiosis

In contrast to the physiological mechanisms described above, mastitis represents a pathological condition that significantly disrupts the milk microbiome. Mastitis, the most common, economically significant, and costly disease in dairy cattle, is characterized by inflammation of the mammary gland and associated physical and chemical changes in milk, including increased somatic cell counts, clotting, and undesirable color changes.

Mastitis should not be regarded as a uniform disease entity but rather as a multi-species and multifactorial infection, in which microbiome alterations depend on the causative agents, infection severity, and duration. It is most commonly associated with pathogens such as *Staphylococcus aureus*, *Streptococcus agalactiae*, other streptococci, enterococci, coliform bacteria, and, less frequently, *Corynebacterium bovis* [32]. However, infections may involve either single dominant pathogens or polymicrobial communities, and these differences are reflected in distinct microbiome configurations [33,34].

The temporal dimension further complicates the interpretation of mastitis-related microbiome changes. Recovery from mastitis may still be accompanied by prolonged dysbiosis, with some milk samples exhibiting persistent dominance of specific taxa such as *Pasteurella* even one month after clinical recovery, whereas others show gradual restoration of microbial balance within 1–3 months [33,35]. These observations indicate that microbiome recovery trajectories are variable and may depend on both host-related factors and the initial etiological agents.

Taken together, these findings demonstrate that mastitis affects the milk microbiome in a context-dependent manner, forming a continuum of microbiome states shaped by pathogen identity, infection severity, and disease progression. This complexity makes it difficult to generalize its impact on beneficial microorganisms and highlights the need for strain-level and longitudinal analyses to better understand microbiome resilience and recovery.

Recent studies using high-throughput sequencing have shown that mastitis is associated with significant, context-dependent alterations in milk microbial communities rather than a uniform dysbiotic signature across all systems. Farm-level comparisons indicate that the predominant mastitis-associated taxa vary with management and incidence patterns: In dairy farms with differing subclinical mastitis incidence rates, genera such as *Klebsiella*, *Escherichia–Shigella*, and *Streptococcus* were significantly enriched in herds with low incidence, whereas *Streptococcus* and *Corynebacterium* predominated in herds with high incidence [34]. These pathogen associations were accompanied by broader community shifts, including differences in phylum-level composition and clusters of gut-associated taxa that co-occur, suggesting environmental or fecal contamination impacts on the milk microbiota [34,36]. In studies of recurrent *Klebsiella* mastitis, taxa linked to the bovine gut, such as *Faecalibacterium* and Ruminococcaceae, were enriched in affected cows, consistent with increased teat-end exposure to fecal microbes and persistent microbial signatures despite the absence of overt clinical signs at sampling [36]. Importantly, although some states of mastitis are characterized by reduced alpha diversity and the dominance of specific pathogens, this pattern is not universal. Ref. [37] reported that subclinical mastitis is associated with moderate shifts in community composition and decreased diversity relative to healthy controls, yet both clinical and subclinical cases can retain substantial overlap in community structure. Together, these findings emphasize that mastitis-associated dysbiosis reflects a continuum of community alterations shaped by pathogen identity, farm ecology, and clinical presentation rather than a single stereotyped microbial signature.

While dairy cows also commonly experience other conditions, such as bovine respiratory disease or metabolic disorders, including subacute ruminal acidosis, ketosis, and hypocalcemia, studies of [38,39] addressing these diseases mainly focus on chemical composition or milk yield rather than microbiome composition. Consequently, existing reviews concentrate on mastitis-related changes in the milk microbiome, leaving a notable gap in knowledge regarding the milk microbiome in cows affected by other diseases or health conditions and highlighting the need for broader investigations encompassing diverse disease contexts [16,18].

## 4. Non-Host Factors Shaping the Bovine Milk Microbiome

In addition to host-related determinants, the milk microbiome is also shaped by external factors, including environmental conditions, housing systems, and technological processes. Although these factors do not originate from the animal, they play a key role in determining the baseline microbial composition of milk and may act as sources of contamination or selective pressures during milk handling and processing.

### 4.1. Housing Environment

The microbiota colonizing post-milking milk primarily originates from the cow’s skin, which is itself shaped by the rearing environment. Studies have shown that bacterial load and somatic cell count (SCC) on teat skin are higher in cows with contaminated legs and udders. Moreover, taxa commonly identified in milk, such as *Propionibacterium* spp., *Corynebacterium* spp., and *Staphylococcus* spp., were not detected in samples collected directly from the milk cistern, suggesting their entry into milk post-milking or during sample collection [12]. Other research highlights the role of skin and milking parlor air as significant contributors to milk microbial communities and suggests that the prevalence of Bacteroidetes, *Corynebacterium*, *Pseudomonas*, Moraxellaceae, and *Staphylococcus* may be overestimated [40].

Recent findings also underscore the importance of housing conditions. Milk from grazing cows appears to contain higher proportions of environmental bacteria, such as Pseudomonas, Lactococcus, and Flavobacterium, whereas milk from indoor-housed cows is enriched in gut-associated taxa, including Ruminococcus, Ruminococcaceae, Eremococcus, and Prevotella. These studies further highlight the critical influence of teat skin and fecal contamination as primary sources shaping milk microbial communities [41]. Additionally, host genetics, mastitis control practices, herd size, stocking density, and seasonal factors such as temperature and humidity at the time of collection further modulate both microbial load and composition [6,16,42].

Although housing conditions clearly influence the composition of milk microbial communities, their impact on milk quality or pathogenic potential remains unclear. Nonetheless, considering these differences may be beneficial in dairy processing, particularly for raw milk–based fermented products, where the resident microbes can play a critical role in shaping texture and flavor.

### 4.2. Milking Infrastructure

The infrastructure of dairy farms can serve as a significant source of milk contamination. Genomic analyses of material collected from filtration systems have revealed the presence of bacteria belonging to various genera, including *Acinetobacter*, *Enterobacter*, *Bifidobacterium*, *Bacillus*, *Corynebacterium*, *Pseudomonas*, *Escherichia*, *Lactococcus*, *Ralstonia*, *Staphylococcus*, and *Bradyrhizobium* [43,44]. Storage tanks and components of milking equipment can facilitate the formation of biofilms, which are complex microbial communities embedded in an extracellular matrix. These biofilms may harbor bacteria such as *S. aureus*, *P. aeruginosa*, *K. pneumoniae*, and *K. oxytoca*, as well as molds and yeasts. Even with routine disinfection, biofilms may persist on equipment surfaces. Therefore, maintaining strict hygiene standards is crucial, not only through disinfection but also by using anti-adhesive agents, ensuring proper temperature control, and maintaining adequate flow of cleaning solutions throughout the milking installation [45,46].

### 4.3. Post-Milking Milk Processing

The technological chain involved in milk collection, transport, and processing plays an increasingly recognized role in shaping the milk microbiome and its derivatives. Ref. [4] examined the microbiota along the transport and storage chain of raw milk and observed substantial changes in microbial composition at different stages. Raw milk was dominated by *Kocuria*, *Dermacoccus*, *Dietzia*, *Lactococcus*, *Clostridium XI*, *Enhydrobacter*, and *Psychrobacter.* In bulk tanks, anaerobes such as *Bacteroides*, *Parabacteroides*, *Veillonella*, *Roseburia*, and *Lachnospiraceae* were identified, likely originating from the milking system. After storage and transport, Proteobacteria, mainly *Pseudomonas*, *Psychrobacter*, and *Acinetobacter*, became predominant. Some taxa, such as Actinobacteria and Acidobacteria, which were absent at earlier stages, likely originated from the storage and transport tanks themselves.

Ref. [5] analyzed microbiome changes during raw milk pasteurization from storage tanks to final products such as skimmed milk and cream. Their findings showed that during separation, filtration, pasteurization, and standardization, both the number and diversity of bacteria changed dynamically. Genetic analyses of isolates grown on plate count agar (PCA) revealed that *Aerococcus*, *Acinetobacter*, *Enterococcus*, *Pseudomonas*, *Staphylococcus*, *Streptococcus*, *Weissella*, *Kocuria*, *Corynebacterium*, and *Jeotgalicoccus* were the dominant genera in raw milk. After separation, cream was dominated by *Jeotgalicoccus*, *Corynebacterium*, *Kocuria*, *Acinetobacter*, and *Aerococcus*, while the remaining taxa prevailed in skimmed milk. Raw cream exhibited greater microbiome diversity than skimmed milk. Pasteurization did not significantly change bacterial counts in skimmed milk, while cream showed a 6-fold reduction. Following microfiltration, most skimmed milk permeate samples contained no detectable bacteria, with only one sample showing 2.3 log CFU/mL. Standardization reduced the overall bacterial count and altered microbial composition: Populations of Peptostreptococcaceae, Microbacteriaceae, and Pseudomonadaceae decreased, while *Rheinmeria*, *Acinetobacter*, *Streptococcus*, Lachnospiraceae, and Bacillaceae remained stable or increased.

A study comparing the microbiome of pre-pasteurized and pasteurized bovine milk demonstrated that pasteurization significantly reduced bacterial counts from 2.17–8.89 log CFU/mL to 0.20–3.97 log CFU/mL. *Bacillus* spp., including heat-resistant species, were detected in pre-pasteurized milk but were not observed after pasteurization. Interestingly, researchers identified genera such as *Hydrogenophaga*, *Undibacterium*, *Thermomonas*, *Diaphorobacter*, *Hydrogenophilus*, *Macellibacteroides*, *Dermacoccus*, *Sphingobium*, and *Acidovorax* only in post-pasteurization samples. None of these are recognized as heat-resistant, suggesting that their presence likely reflects post-pasteurization contamination [47]. Conversely, other studies report that heat-resistant bacteria, including *Bacillus cereus*, *Bacillus sporothermodurans*, and *Geobacillus stearothermophilus*, can persist even in UHT milk. In one survey, these taxa were detected in 38 of 100 commercially available milk samples from the Brazilian market, highlighting the importance of monitoring the milk microbiome even after high-temperature processing [48].

Ref. [49] also emphasized the dynamic nature of the microbiome in the transport and processing chain of milk intended for powder production. Samples collected from bulk tanks and tanker trucks during two lactation periods showed high diversity dominated by *Pseudomonas*, *Acinetobacter*, *Lactococcus*, and *Corynebacterium*. Milk from dairy plant storage silos exhibited lower taxonomic diversity due to the dominance of psychrotrophic spoilage-associated bacteria (*Pseudomonas*, *Acinetobacter*) and non-psychrotrophic spoilage-associated *Lactococcus*. Pasteurized and separated milk showed higher microbial diversity than whole milk storage, with *Lactococcus*, *Acinetobacter*, and *Streptococcus* as dominant taxa. After drying, taxonomic diversity decreased in mid-lactation milk and increased in late-lactation milk. The dominant bacteria in the final powder differed: Mid-lactation milk powder was dominated by thermophilic *Thermus*, *Geobacillus*, and *Streptococcus*, whereas late-lactation milk powder contained predominantly psychrotrophic *Pseudomonas* and *Acinetobacter*.

Milk microbiome undergoes substantial shifts during the production of fermented products. During kefir fermentation, inoculation with kefir grains rapidly restructures the microbial community, with significant changes observed as early as 8 h. As reported by [50], an initially diverse milk microbiome, comprising multiple taxa with relative abundances above 1%, shifts toward a more homogeneous community dominated by *Lactobacillus*, *Leuconostoc*, and *Acetobacter*, together accounting for over 98% of the total population. Although Lactobacillus initially predominates, its dominance decreases after 24 h in favor of *Leuconostoc* and *Acetobacter*. Notably, the initially abundant *Pseudomonas* (17%) is markedly reduced during fermentation, which is relevant from a food safety perspective. Similar trends in microbiome restructuring following kefir grain inoculation have been reported by [51], indicating that dominance within kefir grains does not directly translate to dominance during fermentation.

Comparable microbial dynamics are observed during cheese production. In smear-ripened cheeses, the initially diverse milk microbiome shifts rapidly after inoculation toward dominance of *Lactococcus* and *Leuconostoc*, which together account for approximately 99% of the microbiota during the first days of ripening. However, the relative abundance of lactic acid bacteria (LAB) declines substantially over time, reaching as low as ~3% by the end of ripening. Concurrently, environmental and nonstarter taxa increase, with *Psychrobacter* becoming dominant (up to 78%) and *Pseudoalteromonas* reaching 15–27% during later stages of ripening [52]. The emergence of such taxa may indicate deterioration in quality or environmental contamination.

The mentioned studies show that changes in the milk microbiome occur at each technological stage. Every processing step may serve as a potential source of contamination by both pathogenic and non-pathogenic bacteria, creating environmental niches for psychrotrophs, such as *Pseudomonas* and *Acinetobacter*, during refrigeration, or for thermophiles, such as *Thermus* and *Geobacillus*, during heat treatments. Moreover, processes such as pasteurization may not only eliminate most bacteria in raw milk but also favor the survival of spore-forming, heat-resistant taxa. These observations reflect a clear microbial succession, confirming the importance of proper hygiene practices and the control of heat-related processes, and highlighting the significance of hurdle technology in ensuring food safety. There is an emerging need for testing milk-derived food with high-throughput sequencing at each step of processing and at least at the end of the planned shelf life of these products, which can help understand these microbiome shifts, which are crucial for improving milk processing strategies, extending product shelf life, and minimizing post-pasteurization contamination (Table 1).

## 5. Conclusions

The milk microbiome is a dynamic ecosystem strongly influenced by cow health, particularly mastitis, which can persistently alter microbial balance even after clinical recovery. Environmental and technological factors, including farm hygiene, milking infrastructure, and processing, further shape microbial communities, highlighting the importance of strict hygiene and monitoring throughout the dairy chain. Despite progress in molecular analyses, the core milk microbiome remains poorly defined, and the impact of other bovine diseases is largely unknown. Future research should focus on standardized, longitudinal studies to clarify interactions between host, environment, and microbes, providing a foundation for safer milk production and microbiome-informed herd management strategies.

## Figures and Tables

**Table 1:** Dynamics of the bovine milk and milk products microbiome across production and processing stages.

Milk/Product Stage	Key Bacterial Taxa	Notes/Dynamics	References
Raw milk (healthy cows)	*Faecalibacterium*, *Lachnospiraceae*, *Propionibacterium*, *Aeribacillus*, *Pseudomonas*, *Ralstonia*, *Psychrobacter*, *Bacillus*, *Enterococcus*, *Lactococcus*, *Halomonas*, *Clostridium*, *Staphylococcus*, *Lysinibacillus*, *Streptococcus*, *Marinilactibacillus*, *Lactobacillus*, *Vagococcus*, *Alkaliphilus*, *Macrococcus*	Highly variable; influenced by udder health, lactation stage, and sampling method	[11,13,14,15,16,17]
Bulk tank/post-transport raw milk	*Kocuria*, *Dermacoccus*, *Dietzia*, *Lactococcus*, *Clostridium XI*, *Enhydrobacter*, *Psychrobacter*	Anaerobes such as *Bacteroides*, *Parabacteroides*, *Veillonella*, *Roseburia*, *Lachnospiraceae* appear; environmental contribution from milking system	[4]
Storage tanks/transport	*Proteobacteria* (*Pseudomonas*, *Psychrobacter*, *Acinetobacter*), *Actinobacteria*, *Acidobacteria*	Shift toward psychrotrophic spoilage-associated bacteria	[4,49]
Skimmed milk	*Acinetobacter*, *Streptococcus*, *Lachnospiraceae*, *Bacillaceae*	Pasteurization reduces total counts; some taxa remain stable	[5,47,49]
Cream	*Jeotgalicoccus*, *Corynebacterium*, *Kocuria*, *Acinetobacter*, *Aerococcus*	Higher diversity than skimmed milk; 6-fold reduction after pasteurization	[5]
Pasteurized milk	*Hydrogenophaga*, *Undibacterium*, *Thermomonas*, *Diaphorobacter*, *Hydrogenophilus*, *Macellibacteroides*, *Dermacoccus*, *Sphingobium*, *Acidovorax*	Likely post-pasteurization contamination; total bacterial counts significantly reduced	[47]
UHT/high-heat processed milk	*Bacillus cereus*, *Bacillus sporothermodurans*, *Geobacillus stearothermophilus*	Heat-resistant spore-formers can persist	[48]
Kefir fermentation	*Lactobacillus*, *Leuconostoc*, *Acetobacter*	Initial dominance by *Lactobacillus*; shifts toward *Leuconostoc* and *Acetobacter* over 24 h; initial *Pseudomonas* reduced	[50,51]
Smear-ripened cheese	*Lactococcus*, *Leuconostoc*, *Psychrobacter*, *Pseudoalteromonas*	LAB dominate initially (~99%); decline over ripening; environmental/nonstarter taxa increase later	[52]

## Data Availability

No new data were created or analyzed in this study. Data sharing is not applicable to this article.
